# Modeling mutation-specific arrhythmogenic phenotypes in isogenic human iPSC-derived cardiac tissues

**DOI:** 10.1038/s41598-024-52871-1

**Published:** 2024-01-31

**Authors:** Thomas L. Maurissen, Masahide Kawatou, Víctor López-Dávila, Kenji Minatoya, Jun K. Yamashita, Knut Woltjen

**Affiliations:** 1 Department of Life Science Frontiers, Center for iPS Cell Research and Application (CiRA), Kyoto University, Kyoto, 606-8507 Japan; 2 Department of Cell Growth and Differentiation, Center for iPS Cell Research and Application (CiRA), Kyoto University, Kyoto, 606-8507 Japan; 3 Department of Cardiovascular Surgery, Kyoto University Graduate School of Medicine, Kyoto, 606-8507 Japan; 4 Present Address: Roche Pharma Research and Early Development, Cardiovascular, Metabolism, Immunology, Infectious Diseases and Ophthalmology, Roche Innovation Center Basel, F. Hoffmann-La Roche Ltd., Basel, Switzerland; 5 Present Address: Gourmey, Paris, France; 6 Present Address: Department of Cellular and Tissue Communications, Graduate School of Medicine, University of Tokyo, Tokyo, Japan

**Keywords:** Induced pluripotent stem cells, Arrhythmias, Cardiovascular models, CRISPR-Cas9 genome editing

## Abstract

Disease modeling using human induced pluripotent stem cells (hiPSCs) from patients with genetic disease is a powerful approach for dissecting pathophysiology and drug discovery. Nevertheless, isogenic controls are required to precisely compare phenotypic outcomes from presumed causative mutations rather than differences in genetic backgrounds. Moreover, 2D cellular models often fail to exhibit authentic disease phenotypes resulting in poor validation in vitro. Here we show that a combination of precision gene editing and bioengineered 3D tissue models can establish advanced isogenic hiPSC-derived cardiac disease models, overcoming these drawbacks. To model inherited cardiac arrhythmias we selected representative N588D and N588K missense mutations affecting the same codon in the hERG potassium channel gene *KCNH2*, which are reported to cause long (LQTS) and short (SQTS) QT syndromes, respectively. We generated compound heterozygous variants in normal hiPSCs, and differentiated cardiomyocytes (CMs) and mesenchymal cells (MCs) to form 3D cardiac tissue sheets (CTSs). In hiPSC-derived CM monolayers and 3D CTSs, electrophysiological analysis with multielectrode arrays showed prolonged and shortened repolarization, respectively, compared to the isogenic controls. When pharmacologically inhibiting the hERG channels, mutant 3D CTSs were differentially susceptible to arrhythmic events than the isogenic controls. Thus, this strategy offers advanced disease models that can reproduce clinically relevant phenotypes and provide solid validation of gene mutations in vitro.

## Introduction

Pre-clinical research requires physiologically relevant disease models that faithfully recapitulate patient pathophysiology and clinical manifestations. A shift towards human-based in vitro cellular models continues to progress, since in vivo animal models often fail to accurately predict treatment responses, resulting in poor success rates for new drugs. Human primary cells isolated from donors or patients are insufficiently expandable resources, especially clinically relevant cells such as cardiomyocytes, neuronal cells and pancreatic beta cells. On the other hand, human induced pluripotent stem cells (hiPSCs) provide an unlimited supply of clinically relevant cells that can be obtained from any individual, and can be potentially derived into any cell type for relevant phenotypic assays^1,2^. Thus, hiPSC-based disease modeling is a promising approach to accelerate the elucidation of pathophysiological mechanisms of human diseases and drug discovery. However, some challenges remain, such as the heterogeneity between hiPSC lines and their differentiated derivatives, and the faithful recapitulation of disease phenotypes using in vitro cell-based models.

Varying genetic backgrounds amongst iPSC lines indeed resulted in phenotypic variability that can confound data interpretation^3^. This is especially valid for hiPSC-based models generated for monogenic diseases, where individual patient phenotypes can become undetectable. Such issues associated with genetic differences are circumvented with gene-edited isogenic controls created in well-characterized hiPSC lines from healthy individuals, to obtain both healthy and mutant hiPSC lines with matching genetic backgrounds. The precise introduction or correction of pathogenic mutations in hiPSC lines has been greatly facilitated by CRISPR-Cas9 through HDR-mediated gene editing^4,5^, base editing^6,7^ and prime editing^8^. For pre-clinical research, isogenic hiPSC-based disease modeling enables generating clinically relevant cell types that are only distinguishable by the presumed causative mutations.

Cardiac arrhythmic disorders were among the first to be modeled in vitro with patient-specific hiPSC lines^9–12^, and with isogenic mutant hiPSC lines^13,14^. Differentiation into hiPSC-derived cardiomyocytes (hiPSC-CMs) provided a valuable tool to investigate patient- and mutation-specific disease mechanisms^15,16^. Cellular models of hiPSC-CMs recapitulated cellular phenotypic variations of monogenic diseases with early onset, such as QT prolongation^17^, but often failed to exhibit actual phenotypes of complex arrhythmia syndromes and resulted in poor validation in vitro. 3D cardiac tissue models composed of cardiomyocytes (CMs) and mesenchymal cells (MCs) or other cardiac cell types, however, improved the complexity and relevance of disease-associated phenotypes^18–20^. Among these, we previously developed a drug-induced cardiac arrhythmia model capable of reproducing Torsade de Pointes (TdP)-like waveforms in vitro^21^. As a proof-of-concept for 3D tissue modeling with isogenic iPSCs, we targeted *KCNH2*, encoding the potassium channel Kv11.1 or hERG, where the N588D and N588K mutations uniquely cause two distinct clinical disorders, long (LQTS) and short (SQTS) QT syndromes respectively^22–26^.

In this study, we generate isogenic *KCNH2* N588D and N588K compound heterozygous variants in a fixed hiPSC genomic background, and differentiate cardiomyocytes (iPSC-CMs) and mesenchymal cells (iPSC-MCs) to form cardiac tissues for in vitro phenotyping. Electrophysiological analysis with multielectrode arrays shows repolarization durations consistent with LQTS and SQTS in 2D hiPSC-CM monolayers and in 3D cardiac tissue sheets (CTSs), while pharmacological inhibition of hERG with channel blockers reveals differential susceptibility of mutant 3D CTSs to arrhythmic events. Thus, our strategy combining isogenic gene-edited hiPSCs and 3D tissue engineering offers advanced disease models that are capable of reproducing relevant clinical arrhythmogenic phenotypes and provide solid validation of pathogenic mutations in vitro.

## Results

### Generating isogenic hiPSC mutants

**Figure 1:**
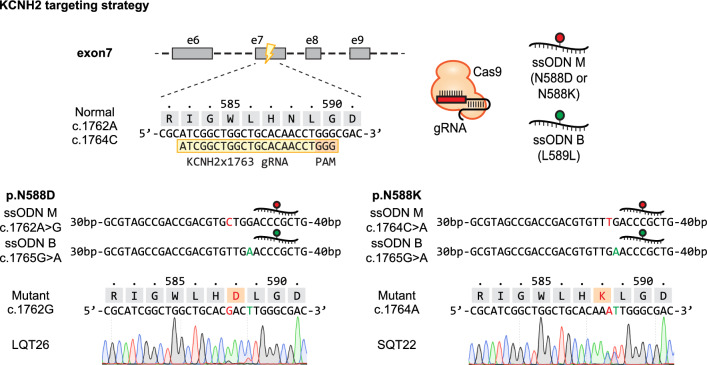
Generation of heterozygous *KCNH2* mutations in hiPSCs. Targeting strategy to independently generate *KCNH2* N588D and N588K missense mutations in the 409B2 hiPSC background with mixed ssODN repair templates, and Sanger sequences of heterozygous mutant clones LQT26 and SQT22. “ssODN M" carries the representative missense mutation, either recreating *KCNH2* c.1762A>G or c.1764C>A, while “ssODN B” generates c.1765G>A, a silent Cas9-blocking mutation.

The N588D and N588K substitutions in *KCNH2* occur in the S5-Pore linker region and result in replacement of a polar Asparagine residue (N) with a negatively charged Aspartic Acid (D) or positively charged Lysine (K)^27^. This results in protein variants that suppress proper IKr channel function. Patients with these mutations are typically heterozygotes or compound heterozygotes, and the mutant proteins are thought to hinder the function of heterotetrameric channel complexes^28,29^. Often, when targeting autosomal genes with CRISPR-Cas9 and single-strand oligonucleotide (ssODN) templates both alleles are edited, leading to homozygous mutations or heterozygous mutations with an undesirable insertion or deletion mutations (indels) on the second allele^5,30^. To obtain heterozygous mutants more reliably, we used a combination of ssODN repair templates carrying the patient missense mutation (ssODN M) or a silent PAM-blocking mutation (ssODN B) preventing Cas9-mediated re-cleavage after editing (Fig. 1). Here, we aimed to independently recreate representative *KCNH2* N588D (LQTS) and N588K (SQTS) mutations in the genomic background of 409B2 hiPSCs^31^, serving as an isogenic baseline for several mutations, since this line is well-established and previously showed high cardiac differentiation potential^32,33^. Leveraging the synergistic effect of cold shock and NHEJ repression that was shown to increase the probability of depositing point mutations from DNA templates by HDR^5^, resulted in the generation of several compound heterozygous clones for L589L and N588D (LQT26, 46, and 79) or N588K (SQT22, 83, 87, and 96; Fig. 1; Supplementary Fig. 1a; Supplementary Table 1). Furthermore, as a byproduct of targeting with ssODN M only or mixed ssODN M and B templates, we also obtained homozygous N588D and N588K mutant clones and homozygous L589L (CTL1, 41, and 60) blocked clones (Supplementary Fig. 1b; Supplementary Table 1). To demonstrate that the silent blocking mutation introduced by ssODN B had no effect on cardiac phenotypes, we included CTL clones as negative controls expected to share similar phenotypes to parental 409B2 controls. Karyotype analysis showed no genomic abnormalities in representative clones (Supplementary Fig. 2).

### HiPSC differentiation and cardiac characterization

**Figure 2:**
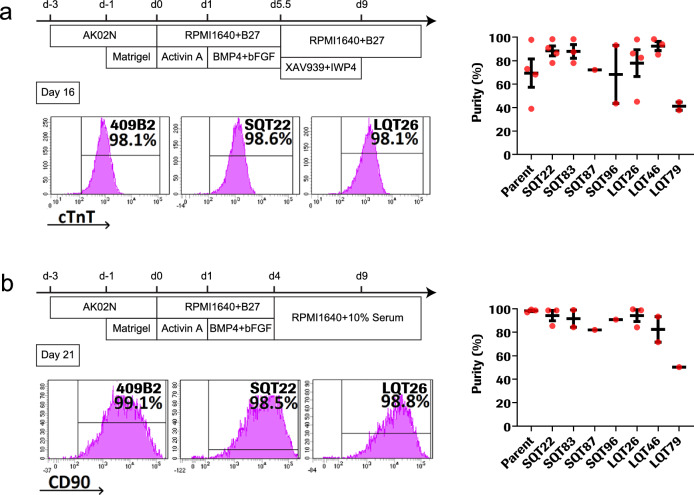
Differentiation and characterization of cardiomyocytes and mesenchymal cells from gene edited hiPSCs. (**a**) Scheme of CM differentiation protocol. Cells were treated with Wnt signal inhibitors (XAV939: 0.25 μM and IWP4: 0.125 μM in RPMI1640 + B27) for 3.5 days, then medium was changed to RPMI1640 + B27 until the end of differentiation. Representative flow cytometry analysis of cTnT at D16 for 409B2 (parent) and SQT22 and LQT26 (mutants) after differentiation. (**b**) Scheme of MC differentiation protocol. Cells were cultured in RPMI1640 + B27 (minus insulin) + 10% FBS from D4. Representative flow cytometry analysis of Thy1 (CD90) at D21 for 409B2 (parent) and SQT22 and LQT26 (mutants) after differentiation. Bar graphs show average purity of cTnT + or Thy1 + (CD90) cells among all differentiation runs and standard deviations. Dots represent independent experiments. Related to Supplemental Table 2.

The 409B2 parent along with heterozygous *KCNH2* LQT and SQT mutant clones were differentiated into hiPSC-derived cardiomyocytes (CMs) and mesenchymal cells (MCs). HiPSC-CM differentiation purity was high (60–80% on average) with variation among independent differentiation runs (Fig. 2a) while hiPSC-MC purity was high and consistent (Fig. 2b; Supplementary Figs. 3–6; Supplementary Table 2). The average percentages of cTnT positive cells was 69.6 ± 24.2% (n = 4) for the 409B2 parent, 70.6 ± 11.5% (n = 9) for the LQT mutants, and 76.2 ± 22.6% (n = 10) for the SQT mutants. In 2D samples with low cardiomyocyte induction efficiency, the cell composition may affect the potential properties. As our differentiation method does not use a strategy of cardiomyocyte enrichment, we excluded the 2D electrical property data for samples with cTnT purity below 65%. Overall, mutations did not change the differentiation capacity.

### Measurement of field potential duration

**Figure 3:**
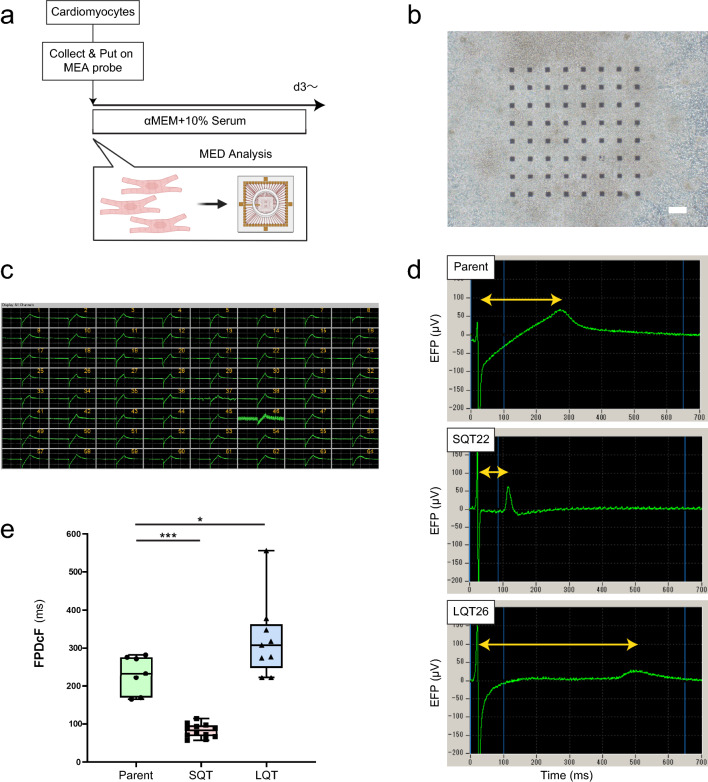
Modeling L/SQTS in 2D on MEA. (**a**) Preparation for Multi-electrode device (MED) Analysis in 2D culture. (**b**) Representative microscopic bright field appearance of 2D cardiomyocytes on MEA electrode (64ch). (**c**) Representative field potential waveforms of 2D cardiomyocytes on MEA electrode (64ch). (**d**) Representative field potential waveform of 2D cardiomyocyte from gene edited hiPSCs (Parent vs SQT vs LQT). (**e**) FPDcF quantification Parent/SQT/LQT (box plot, statistical analysis). Parent (n = 7), SQT22 (n = 12), LQT26 (n = 9), Dunnett’s test *P < 0.05, **P < 0.01 and ***P < 0.001. ”n” is the number of replicate probes used for each clone. Scale bars: 150 μm in (**c**).

Proceeding to phenotyping, we formed hiPSC-CM 2D cultures of the parent and mutant lines on top of multielectrode arrays (MEAs) and measured the extracellular field potential (EFP) for all hiPSC-CM conditions (Fig. 3a,b). EFP recordings revealed synchronized uniform waveforms under spontaneous beating (Fig. 3c). The field potential duration (FPD) corresponds to the QT interval in an electrocardiogram (ECG)^34^. The field potential duration collected by Fridericia’s formula (FPDcF) was significantly shortened for the SQT mutants (SQT22, 82 ± 18 ms, n = 12), and significantly prolonged for the LQT mutants (LQT26, 323 ± 21 ms, n = 9) compared to the 409B2 control group (231 ± 24 ms, n = 7, p < 0.05) (Fig. 3d,e; Supplementary Fig. 7; Supplementary Table 3). Additionally, we confirmed that CTL clones had an FPDcF similar to the parent (Supplementary Fig. 7). These results are consistent with clinical arrhythmogenic phenotypes of LQTS and SQTS respectively.

We previously showed that 2D cultures consist of 1–2 cell layers while 3D CTSs consist of 5–6 cell layers, and 3D structure and cellular heterogeneity were essential requirements to reproduce Torsade de Pointes (TdP)-like waveforms in vitro^21^. Since 3D tissue modeling was expected to have high translatability and relevance for clinical disease phenotypes, arrhythmogenic responses were further investigated in a 3D CTS configuration.

Following 2D phenotyping of all hiPSC-CM lines, we mainly focused on the 409B2 control, and representative SQT22 and LQT26 mutant clones for complex 3D phenotyping, based on the previous CM differentiation efficiencies (409B2: 69.6% cTnT, 98.3% Thy1; SQT22: 88.5% cTnT, 94.2% Thy1; LQT26: 78.1% cTnT, 94.2% Thy1) and FPD recordings.

### Generation of arrhythmia models and response to channel blockers

**Figure 4:**
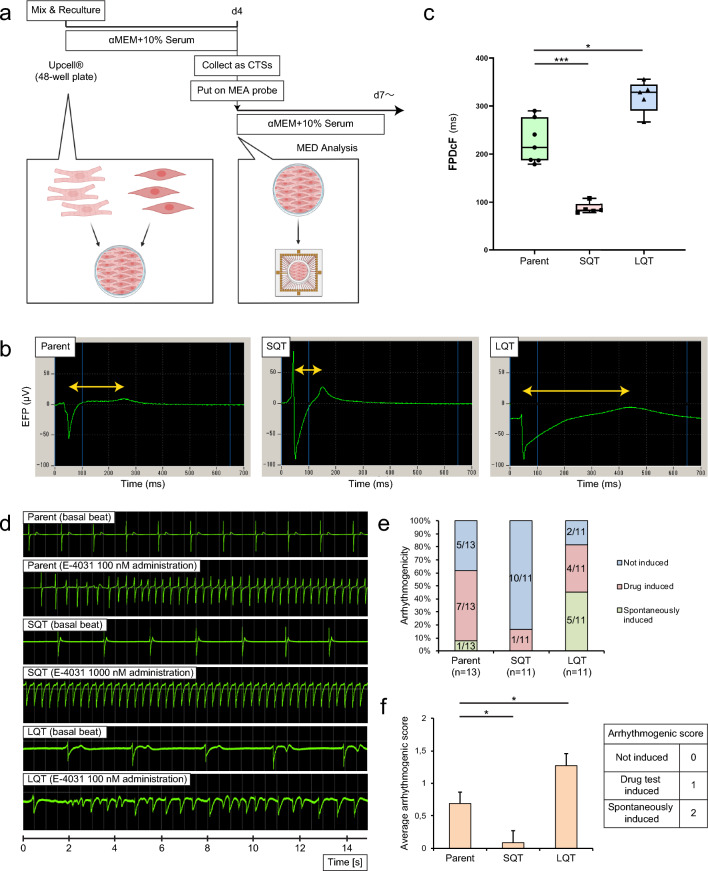
Modeling L/SQTS in 3D CTSs on MEA. (**a**) Generation and characterization of 3D CTSs. (**b**) Representative field potential waveform of 3D cardiac tissue sheet from gene edited hiPSCs (Parent vs SQT22 vs LQT26). (**c**) FPDcF quantification Parent/SQT/LQT (box plot, statistical analysis). Parent (n = 7), SQT22 (n = 5), LQT26 and LQT46 (n = 5), Dunnett’s test *P < 0.05, **P < 0.01 and ***P < 0.001. (**d**) Representative basal and arrhythmic field potential waveform of 3D CTSs in response to IKr blocker (E-4031) (Parent vs SQT22 vs LQT26). (**e**) Tendency of arrhythmogenicity in 3D cardiac tissue sheet (Parent (n = 13), SQT22 (n = 11), LQT26 (n = 11)). The denominator is the total number of samples, and the numerator is the number of samples in which the relevant arrhythmia phenomenon occurred. (**f**) Average of arrhythmogenic score in 3D cardiac tissue sheet (± s.e.m, Parent (n = 13), SQT22 (n = 11), LQT26 (n = 11), Dunnett’s test *P < 0.05).

We generated 3D CTSs from each line by mixing corresponding hiPSC-CMs and MCs in suspension and seeding them onto a temperature-sensitive culture dish, taking into account differentiation efficiencies and purities obtained by FACS (Fig. 2). After cells were detached from the dish as cell sheets, tissue shrinking spontaneously generated 3D structures with thickness of about five cell layers or 100 µm, constituting the arrhythmia models (Fig. 4a; Supplementary Fig. 8a). The percentage of cTnT positive cells in this arrhythmia model was 25 ± 6% (n = 4) for the 409B2 control, 41 ± 10% (n = 4) for the SQT22 mutant, and 29 ± 15% (n = 4) for the LQT26 mutant (Supplementary Fig. 8b). Representative field potential waveforms in 3D CTSs clearly showed differences in FPD (Fig. 4b). The recorded FPDcF averaged 225 ± 13 ms (n = 7), 87 ± 16 ms (n = 5) and 320 ± 16 ms (n = 5) respectively, showing significant FPDcF prolongation for the LQT26 mutant compared to the 409B2 control, while the SQT22 mutant showed significant FPDcF shortening (Fig. 4c; Supplementary Table 4).

Next, we observed the arrhythmic response of 3D CTSs to the IKr blocker E-4031. As a result of E-4031 addition, we observed FPDcF prolongation (Supplementary Tables 3 and 4) and TdP-like and ventricular tachycardia (VT)-like waveforms (Fig. 4d). Moreover, we assessed tachyarrhythmia (TdP-like or VT-like waveforms) induction tendencies during E-4031 administration. The LQT26 mutant tended to show more frequent occurrence of spontaneous arrhythmias before E-4031 administration than the control and the SQT22 mutant (Fig. 4e; Supplementary Fig. 8c–e; Supplementary Tables 5 and 6). Next, we calculated the arrhythmia scores and compared their arrhythmogenicity (Fig. 4f). The LQT26 mutant score was significantly higher than the control group, while the SQT22 mutant score was significantly lower.

## Discussion

Patient-derived hiPSCs can be difficult to compare due to differences in their genetic backgrounds^35,36^. The isogenic hiPSC lines generated here varied only by single nucleotides at the target site in *KCNH2* and accurately reproduced characteristic phenotypes of LQTS and SQTS. As a technical limitation, high efficiency gene editing approaches using Cas9 and ssODNs commonly result in the generation of homozygously edited clones, or clones precisely edited on one allele with a mutagenic indel in the second allele^37,38^. Here, we generated compound heterozygous clones using a combination of ssODNs, where one allele contains the patient missense mutation and the second allele carries a silent blocking mutation preventing Cas9 recognition and cleavage, reducing indels. This isogenic gene editing strategy is generally applicable to model monogenic diseases and validate mutation pathogenicity in vitro within otherwise consistent genetic backgrounds.

In addition, we developed in vitro models of cardiac arrhythmia using isogenic gene-edited hiPSC lines cells organized in 2D and 3D cardiac tissues. A recent study by Brandão et al. was successful at prolonging repolarization in hiPSC-CMs in monolayer culture by mutating different areas of the IKr channel and highlighted phenotypical differences among these different mutations^39^. This study supports our hypothesis that complex arrhythmic phenotypes can be generated in vitro when introducing patient mutations in a healthy genetic background. Using our model, we demonstrated that both types of *KCNH2* mutations led to changes in FPD compared to the isogenic control, however, the occurrence of complex arrhythmic phenotypes might still be dependent on external stimuli such as pharmacological treatments or electrical stimulation. In fact, Shinnawi et al. used pacing stimulation for inducing arrhythmia^40^. This is consistent with arrhythmias not being significantly more likely to occur in our in vitro model of SQTS. Triggering arrhythmia in vitro is an improvement compared to core FPD changes when inducing pathophysiological phenotypic differences between the different isogenic hiPSC-CM lines.

Therefore, we induced arrhythmias by administering the IKr blocker E-4031, which prolongs FPD^10,21^. Concentrations up to 30 nM E-4031 prolonged FPD, but higher doses were required to cause arrhythmic events other than QT prolongation. In N588K mutants, the shortened FPD characteristic of SQTS indicated an increased IKr current, thus requiring a higher concentration of IKr blocker to sufficiently prolong FPD and the emergence of arrhythmias. Conversely, in N588D mutants, the prolonged FPD characteristic of LQTS reflected a decreased IKr current, making tachyarrhythmia more likely to occur even prior to E-4031 administration, and resulting in a higher rate of spontaneously induced arrhythmogenicity. The tendency of arrhythmogenicity in the SQTS model after E-4031 treatment was lower than the control. In general, in LQTS and drug-induced QT prolongation, the prolongation of repolarization time causes electrical instability, which can easily induce early afterdepolarization (EAD), resulting in ventricular arrhythmias. We hypothesized that in this SQTS model (SQT22), the induction of QT prolongation by the administration of E-4031 below concentrations of 30 nM (FPDcF: 100 ± 24 ms) may not be sufficient to generate arrhythmia. Originally, the mechanism of ventricular arrhythmogenesis in SQTS has not been fully elucidated. It is thought that spiral wave reentry is difficult to form without accidental extrasystoles in SQTS and therefore electrical stimulation is necessary to induce arrhythmias^41^. Therefore, these results validate the causality of selected pathogenic mutations with arrhythmic phenotypes. Although what triggers ventricular arrhythmia in the clinical patient of SQTS has not yet been determined, this model is expected to be a tool to further elucidate the pathogenesis of SQTS.

In 3D CTSs, we previously showed that arrhythmias are more likely to occur at a CM to non-CM ratio of approximately 1:1^21^. 3D CTSs with high purity CMs prepared by our CM induction methods^42,43^ never showed arrhythmias in our previous study^21^, indicating that co-existence of MCs is essential to induce arrhythmias through the formation of electrical reentry circuits. Furthermore, the distribution of gap junction protein Connexin-43 expression revealed that CM and non-CM heterogeneity is required for meandering of the spiral reentry center^21^. Slower propagation speed with co-existence of MCs compared to pure CMs may also be a factor. Then, we confirmed that there are no significant differences in the final cellular composition of the CTSs generated in each group so that we could assess arrhythmogenicity with an aligned compositional background. We recognize CD90-positive cells as MCs, which we previously identified as positive for Vimentin, αSMA and Calponin, but how these cells influence arrhythmias is a crucial aspect that requires further investigation^21^. Future studies should better characterize non-CM off-target cells.

The process leading to the emergence of TdP in LQTS is a subject of continued investigation. Pre-TdP stages such as EAD remain evaluated in 2D culture formats that might be inadequate to generate representative TdP. Our TdP model of CTSs allows us to confirm the final form of TdP, the emergence of TdP itself. Therefore, we expect that more complex and unknown processes are taking place. An important advantage of our approach is that it retains simple evaluation of the phenotype results. Nevertheless, the complex processes leading up to TdP represent an important direction for future pathophysiological elucidation, and need to be addressed.

In this study, we demonstrated that the combination of isogenic gene edited hiPSC lines and cardiac arrhythmia modeling allowed us to recapitulate relevant phenotypic responses originating from pathogenic mutations that were reported in patient cohorts. Beyond monogenic arrhythmic disorders, compound mutations causing LQTS have been associated with increased severity^44,45^ and remain poorly investigated in vitro. Our approach could be further applied to generate polygenic or compound mutations involved in cardiac arrhythmia.

## Conclusion

In summary, we developed an advanced disease model integrating human iPSC-based single-nucleotide gene editing and 3D tissue modeling that can potentially overcome limitations in hiPSC line heterogeneity and 2D phenotyping. We recreated cardiac ion channel mutations that exhibit different clinical phenotypes and confirmed that isogenic gene-edited hiPSC lines reproduced the corresponding cardiac arrhythmic syndromes in vitro. Electrophysiological analysis of 2D and 3D cardiac tissues validated FPD changes representative of LQTS and SQTS pathophysiology and that mutant 3D cardiac tissue sheets were more susceptible to drug-induced arrhythmias than the isogenic control. Combined disease modeling at the genetic and tissue levels thus confirmed arrhythmogenic phenotypes of pathogenic mutations consistent with clinical observations in patients. Taken together, this advanced disease modeling strategy will improve the accuracy of obtaining relevant pathophysiological phenotypes and success rates of drug discovery.

## Experimental procedures

### Cell culture

HiPSCs, namely 409B2 (RIKENBRC #HPS0076) were maintained in StemFit AK02N medium (Ajinomoto, Cat. No. RCAK02N), on a surface pre-coated with 0.5 mg/mL laminin-511 (Nippi, Cat. No. 892021) in PBS. For passage, cells were dissociated with Accumax (Innovative Cell Technologies, Cat. No. AM105-500), incubated 10 min at 37 °C, and routinely seeded at a density of 1 × 10^3^ cells/cm^2^ in StemFit AK02N medium supplemented with 10 µM ROCK inhibitor Y-27632 (Wako, Cat. No. 253-00513). After 48 h, cells were cultured without ROCK inhibitor. Cells were tested negative for mycoplasma contamination.

### HiPSC gene editing

HiPSCs were targeted as previously described^5^. Briefly, crRNA and tracrRNA (IDT, Alt-R CRISPR-Cas9) were annealed and RNP complexes were formed by mixing 61 pmol gRNA with 61 pmol Cas9 nuclease (IDT, Alt-R S.p. Cas9 Nuclease V3) at a 1:1 gRNA:Cas9 ratio and incubating for 30 min at room temperature (RT). Finally, equal amounts of ssODN repair templates (IDT, Ultramer DNA Oligonucleotides) totaling 82 pmol were added to the preformed RNP complexes in order to generate compound heterozygous mutants. gRNA and ssODN sequences are listed in Supplementary Tables 7 and 8, respectively. 1 × 10^6^ cells resuspended in 50 µl Opti-MEM I reduced-serum medium (Life Technologies, Cat. No. 31985-062) were added to editing reagents and electroporated in a Nepa Electroporation Cuvette 1 mm gap (Nepa Gene, Cat. No. EC-001) using the NEPA21 Electroporator (Nepa Gene) instrument (Poring pulse: 125 V voltage, 2.5 ms pulse length, 50 ms pulse gap, 2 pulses, 10% pulse decay, + orientation; Transfer pulse: 20 V voltage, 50 ms pulse length, 50 ms pulse gap, 5 pulses, 40% pulse decay, ± orientation). Electroporated cells were treated with cold shock (32 °C for 48 h) and an inhibitor cocktail composed of 2 µM NU7441 and 1 µM SCR7 for 48 h in StemFit AK02N medium supplemented with 10 µM ROCK inhibitor. Afterwards, cells were cultured in StemFit AK02N medium without ROCK inhibitor until approximately 80% confluent.

### Clonal analysis

Cells were harvested 7 days after EP and 400–800 cells were plated in iMatrix511-coated 6 cm dishes. 10 days after plating, colonies approximately 1 mm in diameter were picked in 5 µl of media under the microscope and transferred to a 96-well plate in StemFit AK02N medium supplemented with 10 µM ROCK inhibitor. Depending on the growth rate of picked clones and upon average confluence across the 96-well plate, cells were then split 1:3 into 3 new 96-well plates. When reaching confluence, 2 plates were harvested, resuspended in STEM-CELLBANKER GMP grade (TAKARA BIO, Cat. No. CB047) and transferred into new 96-well plates for storage at − 80 °C and 1 plate was harvested and transferred to a 96-well PCR plate for genomic DNA extraction. For this, 10 µl QuickExtract DNA Extraction Solution (Epicenter, Cat. No. QE09050) per well was added, followed by 6 min incubation at 65 °C then 2 min at 98 °C and storage at − 30 °C.

### Genotyping

For genomic DNA extraction, target sequences were PCR-amplified with KAPA HiFi HS ReadyMix (Kapa Biosystems, Cat. No. KK2602), amplicons were treated with ExoSAP-IT Express reagent (Thermo Fischer Scientific, Cat. No. 75001) for enzymatic cleanup, and Sanger sequencing was prepared with the BigDye Terminator v3.1 CS Kit (Thermo Fischer Scientific, Cat. No. 4337456). Reactions were then purified by ethanol precipitation and acquired on a 3130xl Genetic Analyzer (Applied Biosystems). Sequence alignments were analyzed with Snapgene (GSL Biotech LLC), and sequence trace files with low base calling confidence were excluded from analyses. Genotyping primers are listed in Supplementary Table 9.

### Karyotyping

Representative mutant clones were seeded at a density of 5 × 10^4^ cells in T-25 flasks, outsourced to Nihon Gene Research Laboratories (Japan), and analyzed before confluence. Each clone showed 50 spreads of 46 chromosomes and none showed banding abnormalities in 20 spreads by Geimsa staining pattern. Parent 409B2 (RIKENBRC #HPS0076) karyotypes are available at the RIKEN BioResource Research Center.

### Cell differentiation

Cell differentiation was performed based on our previously reported methods^21,43^. HiPSCs were collected by incubating them with Versene (#15040066, 0.48 mM EDTA solution; Thermo Fisher Scientific) for 5 min at 37 °C. Versene was carefully aspirated and AK02N containing Y-27632 (10 μM) was added. Cells were detached by tapping, collected, counted and seeded onto matrigel (#354230, Growth factor reduced; 1:60 dilution; Corning)-coated wells at a density of 7.2 × 10^4^ cells/cm^2^ in AK02N (0.52 mL/cm^2^) containing Y-27632 (10 μM) and supplemented with 4 ng/mL bFGF and cultured for 2–3 days before induction until full confluence was achieved. One day before induction (d(− 1)), medium was substituted by AK02N (0.52 mL/cm^2^) supplemented with 4 ng/mL bFGF and matrigel (1:60 dilution). To induce cardiac differentiation, medium was switched to RPMI1640 (#21870092, 2 mM l-glutamine, Thermo Fisher Scientific) medium with 1xB27 supplement minus the insulin (insulin-; A1895601, Thermo Fisher Scientific), further supplemented with 100 ng/mL Activin A (ActA; #338-AC, R&D systems, Minneapolis, MN) on day 0 (d0), for 24 h. The medium was then changed to RPMI1640, 2 mM l-glutamine, with 1 × B27 supplement without insulin containing 10 ng/mL human bone morphogenetic protein 4 (BMP4; #314-BP, R&D) and 10 ng/mL bFGF, and cultured the cells for 3–4.5 days with no medium change, depending on the cell type.

For MC differentiation, medium was switched to RPMI1640 (insulin-, (#A1895601) supplemented with 10% fetal bovine serum (FBS, #59901101, MOREGATE; FBS, #SFBM30-2485, EQUITHCH-BIO, INC.) and 2 mM l-glutamine on d4. The same medium was refreshed every 2–3 days until the end of differentiation. For CM differentiation, medium was switched to RPMI1640 with regular B27 supplement (#17504044, Thermo Fisher Scientific) supplemented with Wnt inhibitors XAV939 (0.25 μM) and IWP4 (0.125 μM) on d5.5 and cultured for 3.5 days. On d9, culture medium was changed to RPMI1640 with B27 supplement alone and refreshed every 2–3 days until the end of differentiation.

### FACS analysis

Cardiomyocyte and mesenchymal cell identity was confirmed using FACS analysis on d15 and d21 respectively. Since differences in CM purity can affect electrophysiological results, differentiated CMs with cTnT purity below 65% were excluded from further phenotyping. Additionally, cardiac tissue sheet (CTS) phenotypes were also confirmed using FACS analysis on CTS generation. Cells and CTSs were dissociated by incubating them in Accumax (Innovative Cell Technologies) for 20 min at 37 °C and stained with the LIVE/DEAD fixable Aqua dead cell staining kit (#L34957, Thermo Fisher Scientific) to remove dead cells. MCs were stained with an anti-PDGFRβ antibody (1:100, #558821, BD Bioscience) labelled with Grn-PE and an anti-Thy1 antibody (BioLegend, San Diego, USA, 1:100) labelled with APC in EDTA at room temperature for 30 min. CMs were first fixed in 4% paraformaldehyde (PFA) for 15 min and washed twice with saponin-EDTA (0.25%, Sigma-Aldrich, St. Louis, USA), following by staining with an anti-cardiac isoform of Troponin T (cTnT) antibody (1:50, #MS-295-P, mouse monoclonal, clone 13–11, Thermo Fisher Scientific) labeled with Alexa-488 using the Zenon technology (#Z25002, Thermo Fisher Scientific) according to the manufacturer’s instructions, diluted in saponin-EDTA for 30 min at room temperature. Cells were then washed twice with EDTA (for MCs) and saponin-EDTA (for CMs), suspended in EDTA and subjected to FACS analysis (10,000 events collected per sample) using the Aria II flow cytometer (BD biosciences, Franklin Lakes, NJ).

### 3D cardiac tissue generation

Cardiomyocytes (d15–d21 after differentiation) were dissociated by incubation with 0.25% trypsin (Thermo Fisher Scientific), and mesenchymal cells (d15–d21 after differentiation) were dissociated by incubation with Accumax (Innovative Cell Technologies).

For CTSs consisting of CMs and MCs, the cells were mixed at a 3:1 or 1:1 ratio, and plated onto a 0.1% gelatin-coated 48-multiwell UpCell® at 6.0 × 10^5^ cells/well with 700 μL attachment medium [AM; alpha minimum essential medium (αMEM; Thermo Fisher Scientific) supplemented with 10% FBS, 5 × 10^–5^ M of 2-mercaptoethanol, 50 units/mL penicillin and 50 μg/mL streptomycin] containing 50 ng/mL VEGF_165_ and 10 μM Y-27632. After 4 days in culture, the cells were moved to room temperature. Within 30 min, cells detached spontaneously and floated in the medium as 3D CTSs. CTSs that had holes or macroscopic irregularities were excluded from further evaluation. FACS analysis of the CM positivity rate within the CTS was made when the CTS was created from temperature-sensitive culture dishes. One of the samples was sent for FACS analysis to calculate the CM positivity rate and others were placed on the MED probe.

### Measurement of extracellular field potential and drug treatment

We evaluated the phenotype of hiPSC-derived 2D cardiomyocyte and 3D arrhythmia model of cardiac tissue sheets. EFPs were measured with the MED system (Alpha MED Scientific, Osaka, Japan) using a Multi-electrode device (MED) probe with 64 planer 50 μm square microelectrodes arranged in an 8 × 8 grid at 150-μm intervals (MED-P515A).

For 2D hiPSC-CM culture, the probe was sterilized as above and coated with 2.75 μg/cm^2^ fibronectin (BD) before use. A total of 3 × 10^4^ cells in 2 μL of medium was spread onto the MED probe and incubated at 37 °C. After at least 180 min, medium was added, and the hiPSC-CMs were incubated with alpha minimum essential medium (αMEM) supplemented with 10% FBS, 5 × 10^–5^ M 2-mercaptoethanol, 50 units/mL penicillin, 50 μg/mL streptomycin, 50 ng/mL VEGF_165_ and 10 μM Y-27632 for 2 days. Half of the medium was changed every 2 days. Stable spontaneous EFPs were recorded from three days to twenty-one days after the initial placement after their adhesion to the electrodes had been confirmed.

For 3D CTSs, the probe was sterilized with 70% ethanol and ultraviolet irradiation and coated with 0.1% gelatine (Sigma-Aldrich) before use. A 3D CTS was spread onto the MED probe. The medium was aspirated, and the CTS was incubated at 37 °C. The rest of the procedure was performed as described above.

We measured EFPs according to a previous report with some modifications^21^. Samples were equilibrated for at least 30 min in a CO_2_ incubator in 2 mL of fresh medium prior to the measurements. After equilibration, the MED probes were maintained at 37 °C with thermo-control systems and covered with a lid through which the gas was aerated (O_2_:CO_2_:N_2_ = 20%:5%:75%). EFPs from spontaneously beating samples were filtered with a 1–1000 Hz bandpass filter using the MED64 System. FPD was defined as the interval from the first peak (depolarization) to the second peak (repolarization). After recording the basal state, 2 μL dimethyl sulfoxide (DMSO; Wako) was added, and EFPs were recorded for 10 min. Then, the IKr blocker E-4031 (Wako) was added to obtain the target concentrations, and EFPs were recorded in the same manner as the recordings of DMSO treatment. For control, five DMSO concentrations (0, 0.1%, 0.2%, 0.3%, and 0.4%) were verified (Supplementary Table 10). For E-4031, six drug concentrations (0.3 nM, 3 nM, 10 nM, 30 nM,100 nM and 1000 nM) were selected to evaluate dose-dependent effects (Supplementary Table 11). At each concentration, the EFP was recorded for 10 min and the FPD values from the last 30 beats were averaged and used as the dataset for FPD and waveform analysis. The waveform of one arbitrary electrode among the 64 electrodes was selected for analysis. The selection criterion was that the depolarization and repolarization wave peaks are clearly identified according to the previously reported method^46^.

EFPs were processed with MED64 Mobius software (Alpha MED Scientific). Beat rate, the inter-spike interval (ms), and FPD (ms) were measured. FPD was corrected for the beating rate with Fridericia's formula (FPDcF = FPD/[inter-spike interval/1000]1/3).

#### Definition of arrhythmic waveform on EFP

We defined Ventricular Tachycardia (VT)-like or Torsade de Pointes (TdP) -like waveforms as arrhythmic waveform.

Ventricular Tachycardia (VT)-like waveforms satisfied the following criteria.

- A continuous tachycardia more than twice the basal beating rate for this model.

Torsade de Pointes (TdP)-like waveforms satisfied the following two criteria.

- A continuous and characteristic twisting EFP waveform with variation in polarity.
- Continuous changes in the excitation interval corresponding to twisting changes of the EFP waveform.

#### Assessment of arrhythmogenicity and calculations of arrhythmogenic score

To compare arrhythmogenicity, we defined "drug test induced" as those in which E4031 up to 100 nM produced arrhythmias and "not induced" as those in which no arrhythmias occurred. We designated "spontaneously induced" as those in which arrhythmias had already appeared in the culture process before drug administration.

We defined the arrhythmogenic score as 0 points for non-induced, 1 point for drug test-induced, and 2 points for spontaneously induced, and calculated the average score for each group.

### Motion Vector Prediction (MVP) analysis

We used a high-precision live cell motion imaging system (Motion Vector Prediction; Sony, Tokyo, Japan). We recorded the movies in a resolution of 2048 × 2048 pixels and adjusted the frame rates in the range from 18 to 150 images per second. Motion vectors of beating cells and CTSs were calculated using a block-matching algorithm^47^. From the processes of motion detection and analysis, we obtained the deformation speed as positive values. We measured the chronological fluctuation of the motion vector of 262,144 points consisting of 4 × 4 pixels. We visualized the motion amplitude by color mapping all points included in the view field and analyzed the two dimensional propagation of cellular motion.

### Statistical analysis

All data analyses were performed using JMP version 14.0.0 (SAS Institute, Cary, USA). Comparisons among three or more groups were performed using One-Way ANOVA, followed by Dunnett’s test or Tukey’s test. Values are shown as the mean ± SEM. P values < 0.05 were considered significant.

### Supplementary Information

Supplementary Information.

Supplementary Video 1.

## Data Availability

The datasets generated during and/or analyzed during the current study are available from the corresponding author on reasonable request.
