# Supplementary material for: Modeling mutation-specific arrhythmogenic phenotypes in isogenic human iPSC-derived cardiac tissues
Source: Sci Rep. 2024 Jan 31;14:2586. doi: 10.1038/s41598-024-52871-1 (PMC10831092; doi:10.1038/s41598-024-52871-1)
Supplement: Supplementary file 1 — Supplementary Information. [file 41598_2024_52871_MOESM1_ESM.pdf]

SUPPLEMENTARY INFORMATION

Modeling mutation-specific arrhythmogenic phenotypes in isogenic human iPSC-derived cardiac tissues

Thomas L. Maurissen[1,4,6], Masahide Kawatou[2,3,6], Víctor López-Dávila[2,5,6], Kenji Minatoya[3], Jun K. Yamashita[2*], Knut Woltjen[1*]

[1]Department of Life Science Frontiers, Center for iPS Cell Research and Application (CiRA), Kyoto University, Kyoto 606-8507, Japan

[2]Department of Cell Growth and Differentiation, Center for iPS Cell Research and Application (CiRA), Kyoto University, Kyoto 606-8507, Japan

[3]Department of Cardiovascular Surgery, Kyoto University Graduate School of Medicine, Kyoto 606-8507, Japan

[4]Present address: Roche Pharma Research and Early Development, Immunology, Infectious Diseases and Ophthalmology, Roche Innovation Center Basel, F. Hoffmann-La Roche Ltd., Basel, Switzerland

[5]Present address: Gourmey, Paris, France

[6]These authors contributed equally

This file includes:

Supplementary Figures 1-8

Supplementary Movie 1 legend

Supplementary Tables 1-11

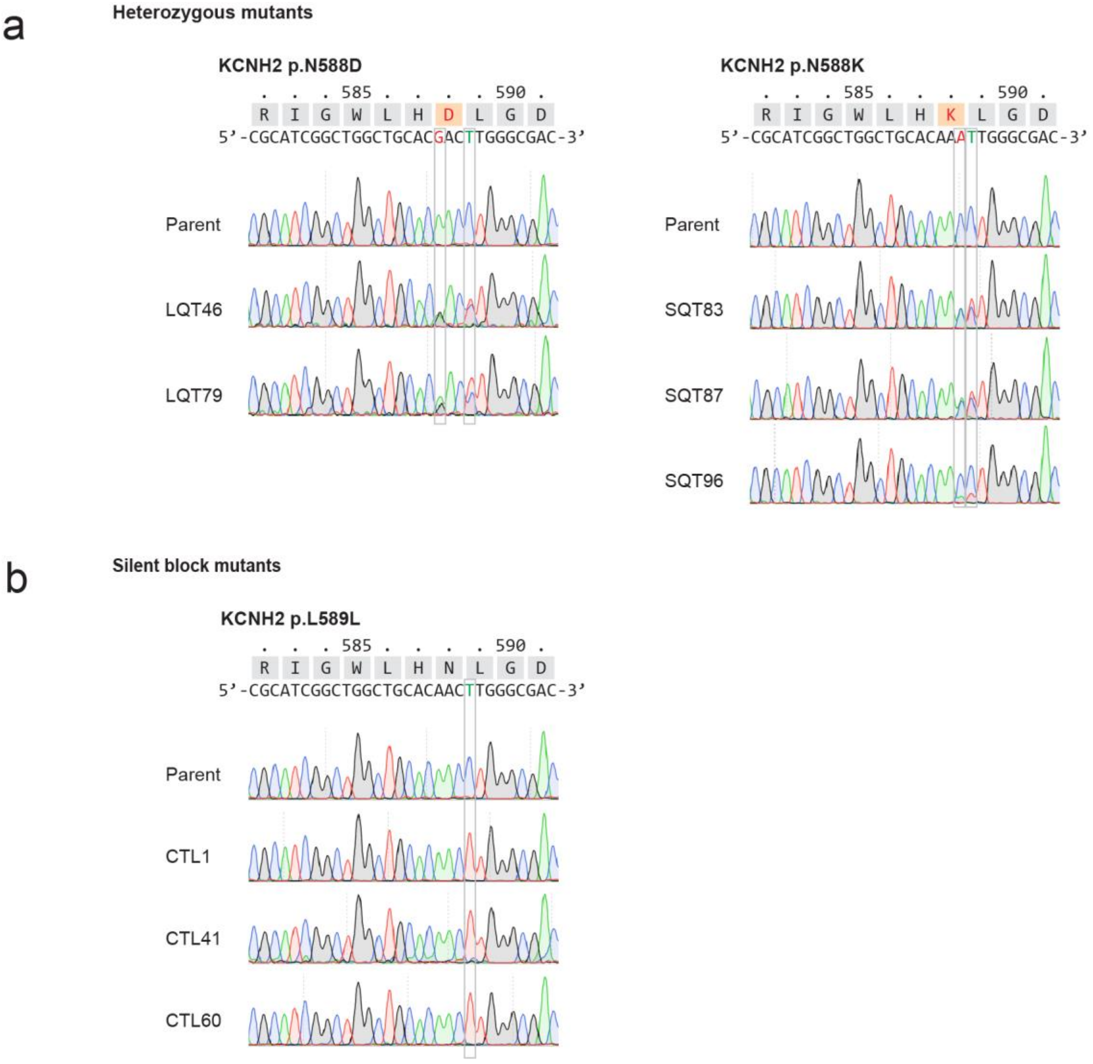

Supplementary Fig. 1 related to Fig. 1: Generation of heterozygous *KCNH2* clones and controls. a Sanger sequences of single clones carrying heterozygous mutations in *KCNH2*, either recreating the N588D (left; LQT46 and LQT79 in addition to LQT26) or N588K (right; SQT83, SQT87 and SQT96 in addition to SQT22) missense mutation. The parent shows the unmodified sequence of the 409B2 hiPSC line. **b** Sequence of *KCNH2* L589L homozygous silent block clones (CTL1, CTL41 and CTL60) obtained when targeting with ssODN M (c.1762A>G) and ssODN B (c.1765C>T).

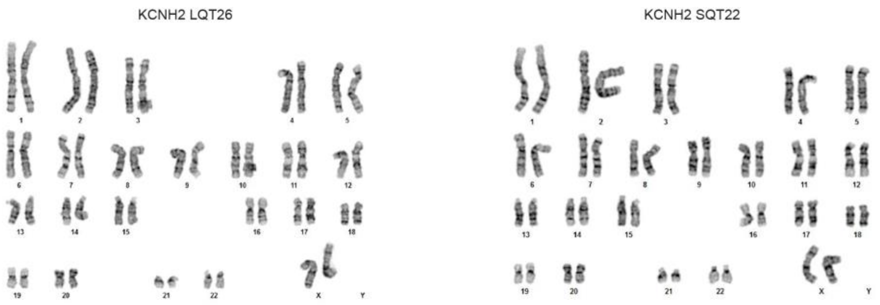

Supplementary Fig. 2 related to Fig. 1: Quality control of established compound heterozygous mutant clones. Karyotypes of KCNH2 LQT26 (left) and SQT22 (right) were analyzed by Giemsa staining (GTG) and 50/50 spreads were normal (46,XX) at 10 passages after targeting the parent 409B2 cell line.

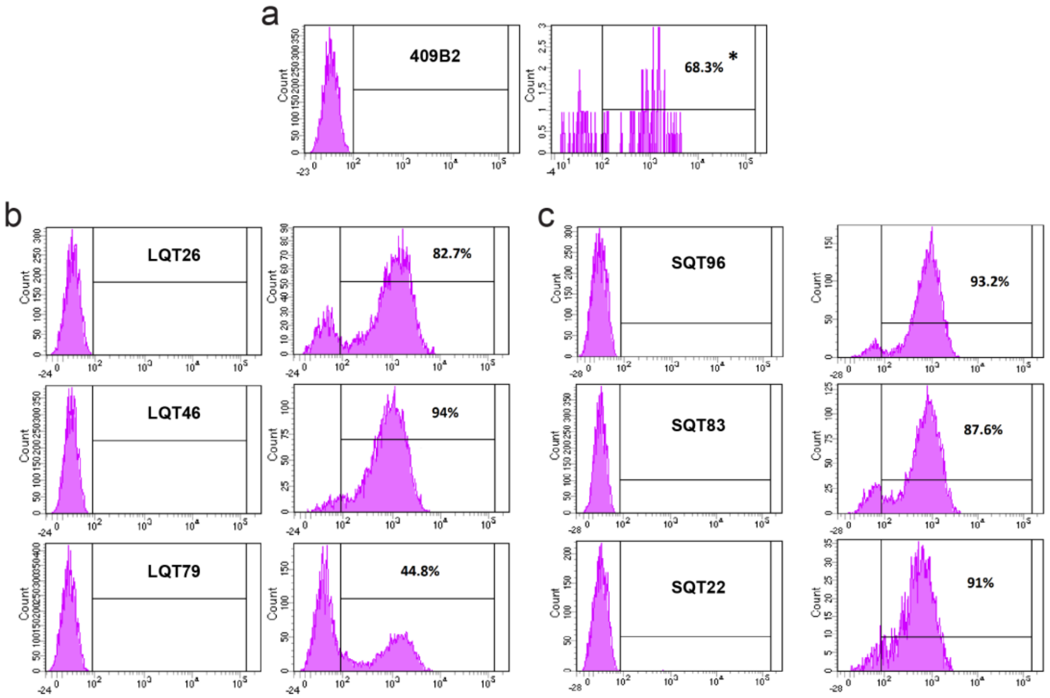

Supplementary Fig. 3 related to Fig. 2: FACS analysis of the first run of differentiation. Histograms are presented in pairs: unstained controls (left) indicate the name of the cell line and cTnT-stained samples (right) indicate cTnT+ cell purity. **a** Parent cell line 409B2. Original analysis was interrupted by an air bubble and indicated above 65% purity (not shown), consistent with the second, limited cell number, reading. **b** Long QT mutants: LQT26, LQT46 and LQT79. **c** Short QT mutants SQT96, SQT83 and SQT22.

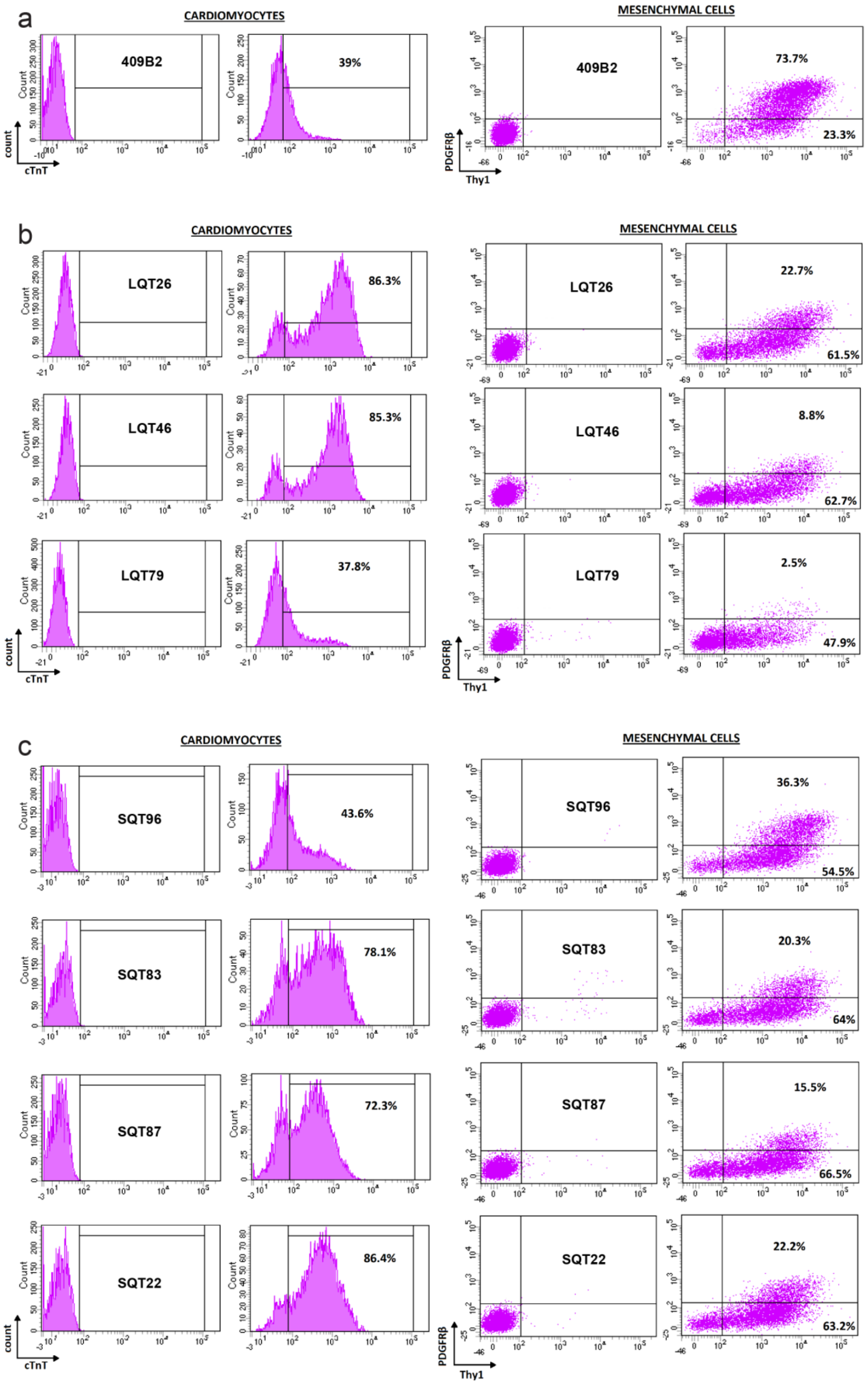

a
CARDIOMYOCYTES
MESENCHYMAL CELLS
409B2
39%
409B2
73.7%
23.3%
b
CARDIOMYOCYTES
MESENCHYMAL CELLS
LQT26
86.3%
LQT26
22.7%
61.5%
LQT46
85.3%
LQT46
8.8%
62.7%
LQT79
37.8%
LQT79
2.5%
47.9%
c
CARDIOMYOCYTES
MESENCHYMAL CELLS
SQT96
43.6%
SQT96
36.3%
54.5%
SQT83
78.1%
SQT83
20.3%
64%
SQT87
72.3%
SQT87
15.5%
66.5%
SQT22
86.4%
SQT22
22.2%
63.2%
count
cTnT
PDGFRβ
Thy1
Count

Supplementary Fig. 4 related to Fig. 2: FACS analysis of the second run of differentiation. Histograms (cardiomyocytes, cTnT+) and scatter plots (mesenchymal cells, Thy1+ and Thy1+/PDGFRβ+) are presented in pairs: unstained controls (left) indicate the name of the cell line and stained samples (right) indicate cell purities. **a** Parent cell line 409B2. **b** Long QT mutants: LQT26, LQT46 and LQT79. **c** Short QT mutants SQT96, SQT83, SQT87 and SQT22.

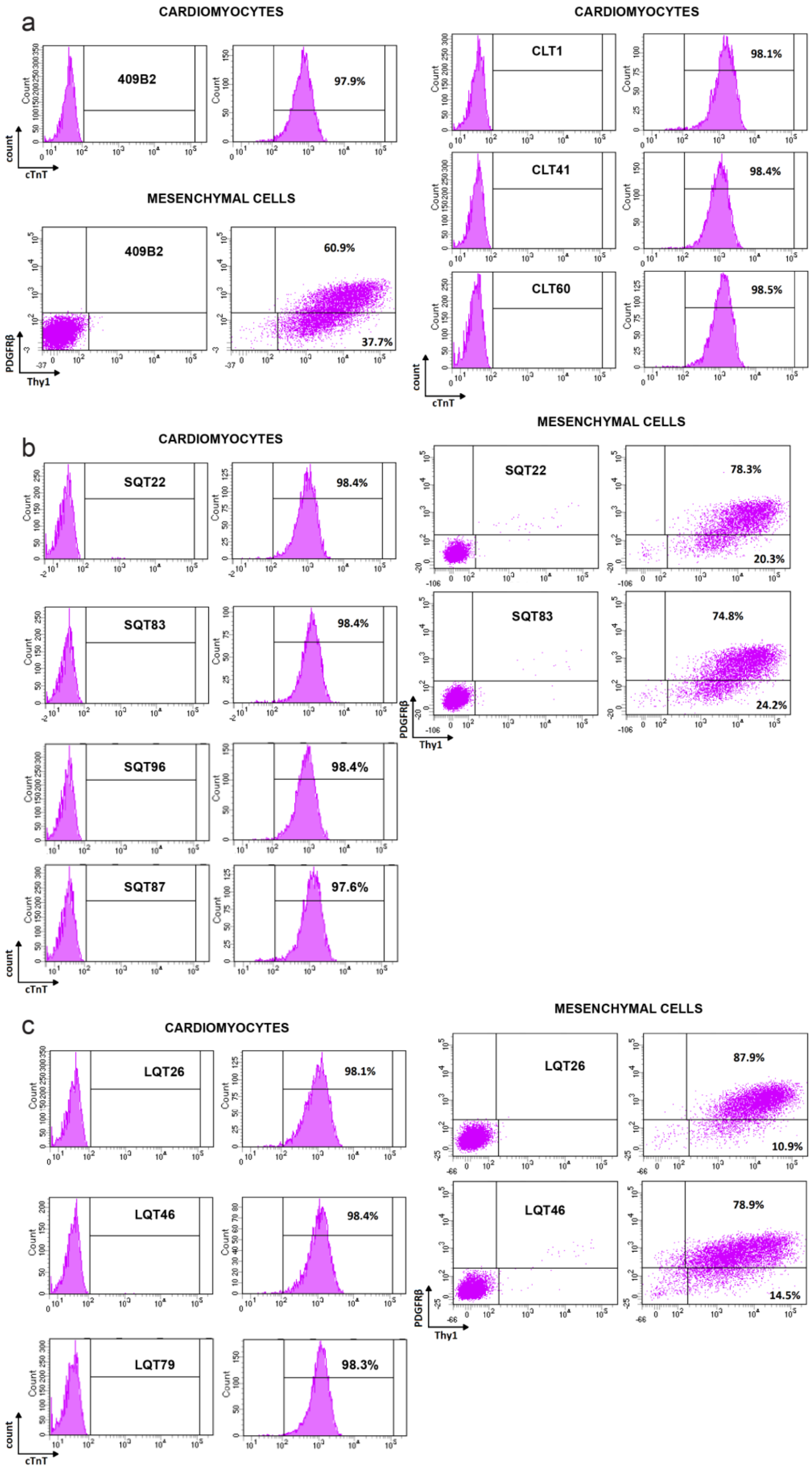
a
CARDIOMYOCYTES
409B2
97.9%
count
cTnT
MESENCHYMAL CELLS
409B2
60.9%
37.7%
PDGFRβ
Thy1
CARDIOMYOCYTES
CLT1
98.1%
CLT41
98.4%
CLT60
98.5%
count
cTnT
MESENCHYMAL CELLS
SQT22
78.3%
20.3%
SQT83
74.8%
24.2%
PDGFRβ
Thy1
b
CARDIOMYOCYTES
SQT22
98.4%
SQT83
98.4%
SQT96
98.4%
SQT87
97.6%
count
cTnT
MESENCHYMAL CELLS
LQT26
87.9%
10.9%
LQT46
78.9%
14.5%
PDGFRβ
Thy1
c
CARDIOMYOCYTES
LQT26
98.1%
LQT46
98.4%
LQT79
98.3%
count
cTnT

Supplementary Fig. 5 related to Fig. 2: FACS analysis of the third run of differentiation. The third run was chosen as the representative experiment for (Figure 2) and (Figure 3). Histograms (cardiomyocytes, cTnT+) and scatter plots (mesenchymal cells, Thy1+ and Thy1+/ PDGFRβ+) are presented in pairs: unstained controls (left) indicate the name of the cell line and stained samples (right) indicate cell purities. **a** Control cell lines: parent cell line 409B2, and block mutations (cardiomyocytes only) CTL1, CTL41 and CTL60. **b** Short QT mutants SQT22, SQT83, SQT96 and SQT87. **c** Long QT mutants LQT26, LQT46 and LQT79.

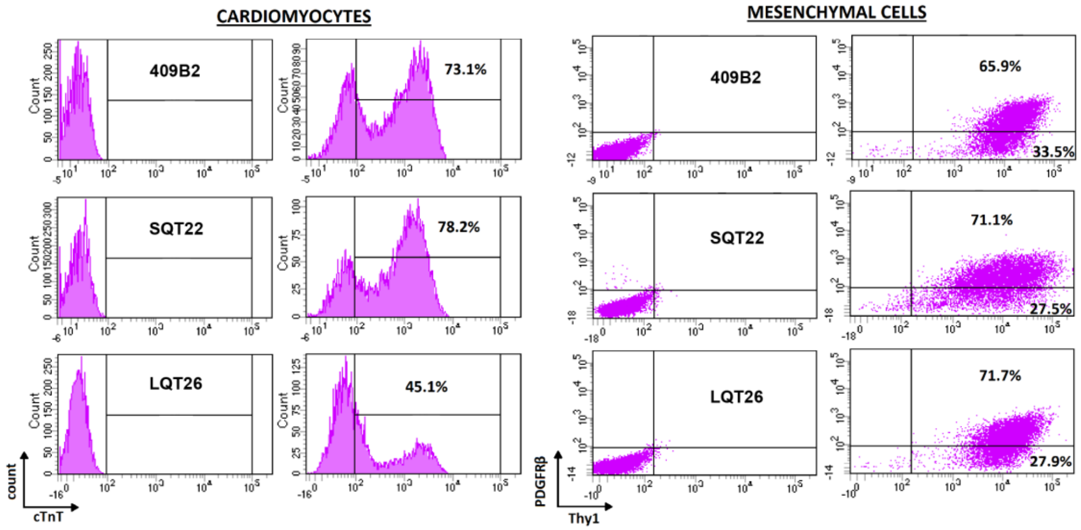

Supplementary Fig. 6 related to Fig. 2: FACS analysis of the fourth run of differentiation. The fourth run was chosen as the representative experiment for (Figure 4), and it consists of the three highest interest cell lines: the parent 409B2, and the mutants SQT22 and LQT26. Histograms (cardiomyocytes, cTnT+) and scatter plots (mesenchymal cells, Thy1+ and Thy1+/ PDGFRβ+) are presented in pairs: unstained controls (left) indicate the name of the cell line and stained samples (right) indicate cell purities.

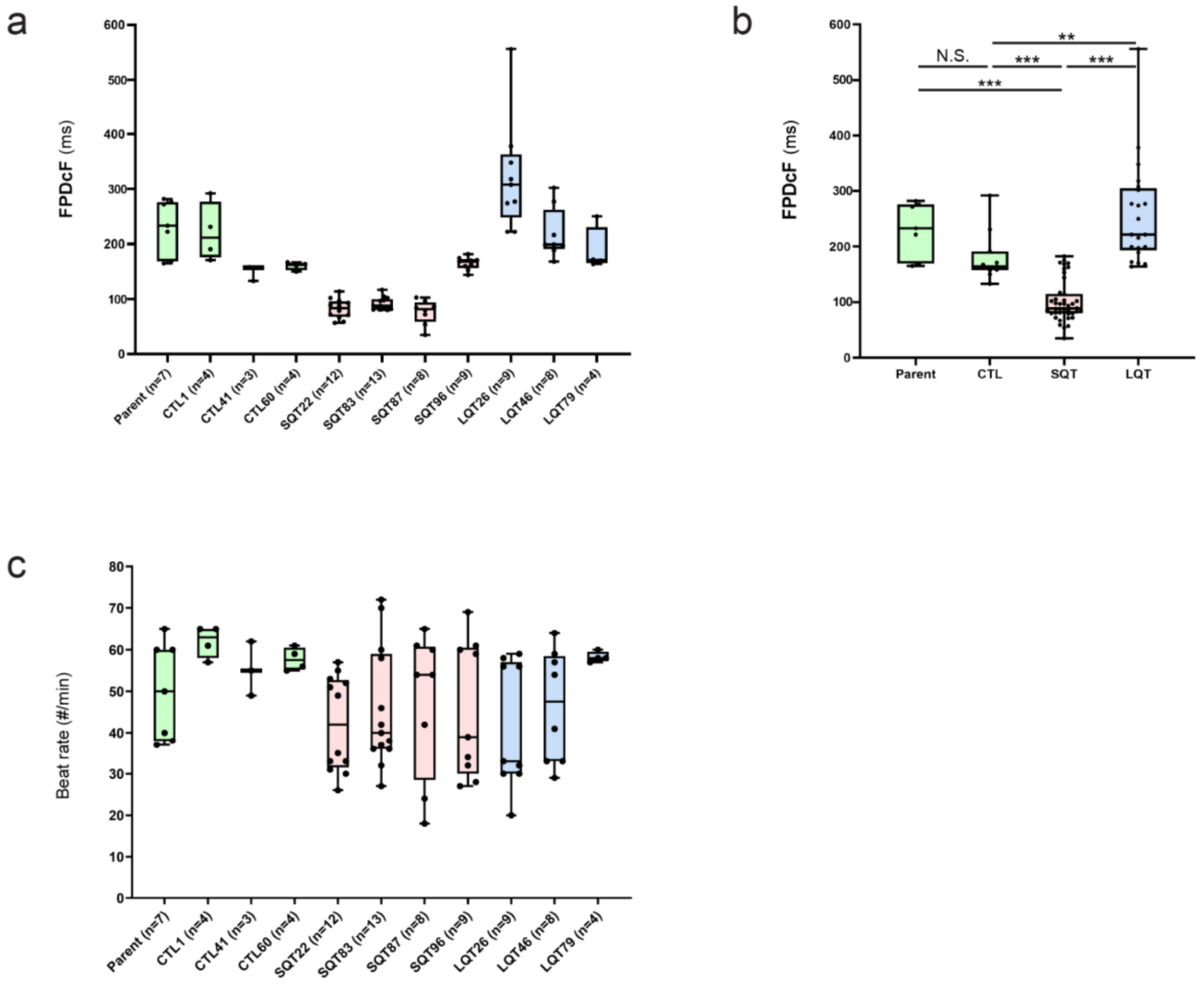

Supplementary Fig. 7 related to Fig. 3: Modeling L/SQTS in 2D on MEA. a FPDcF quantification Parent/SQT/LQT (Box plot). **b** FPDcF quantification Parent/SQT/LQT (Box plot, statistical analysis). Parent (n=7), CTL (n=11), SQT22 (n=42), LQT (n=21), Steel-Dwass's test *P<0.05, **P<0.01 and ***P<0.001. N.S: no significance. **c** Beat rate quantification Parent/SQT/LQT (Box plot).

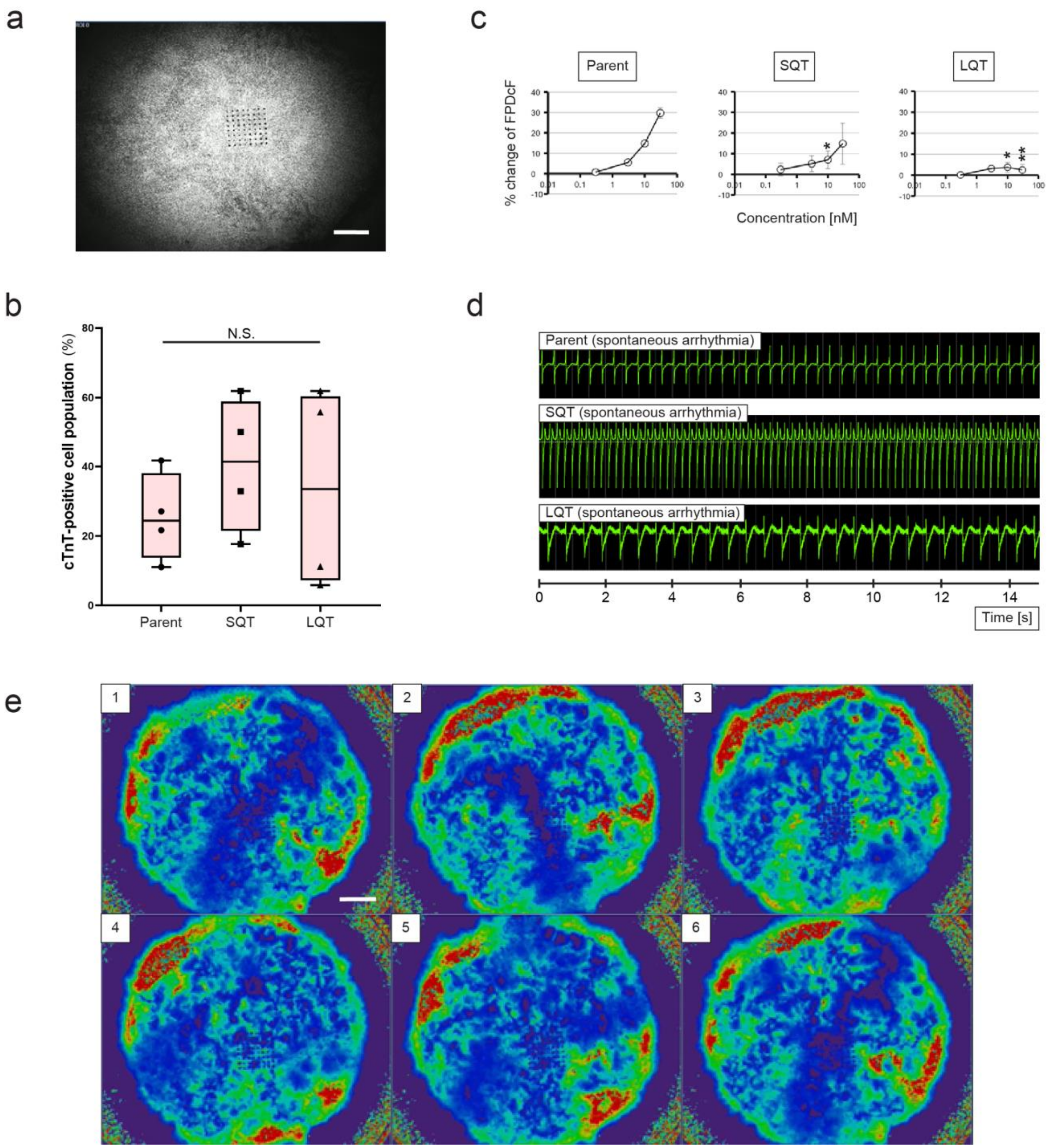

Supplementary Fig. 8 related to Fig. 4: Modeling L/SQTS in 3D CTSs on MEA. a Bright field image of whole cardiac tissue sheet. **b** cTnT-positive cell quantification of 3D CTSs in Parent/SQT/LQT (Box plot, statistical analysis). Parent, SQT22 and LQT26 (n= 4). Dunnett's test. N.S: no significance. **c** Relative change in FPDcF after IKr blocker E-4031 treatment (±s.e.m, Parent (n=3), SQT22 (n=5) and LQT46 (n=3). Dunnett's test *P<0.05 and **P<0.01 compared to Parent in each concentration). **d** Representative

spontaneous arrhythmic field potential waveform of 3D CTSs (Parent vs SQT22 vs LQT26). **e** Representative arrhythmic excitation propagation of 3D CTSs visualized by motion vector prediction (captured from Supplementary Movie 1). (LQT26 CTS, spontaneously induced). Scale bars: 1 mm in A, E.

Supplementary Movie 1:

Representative arrhythmic excitation propagation of 3D CTSs visualized by motion vector prediction, related to Supplementary Figure 8. (LQT26 CTS, spontaneously induced). Scale bars: 1 mm.

Supplementary Table 1 related to Fig. 1: Targeting outcomes obtained following electroporation with different donor templates (ssODN1 and ssODN2) and treatment conditions (cold shock and NHEJ inhibitors).

Experimental conditions				Gene editing outcomes						
Treatment	Mutation	ssODN1	ssODN2	M/M	M/B	M/WT	B/B	Indel	WT	n
32°C + N	N588D	M	-	1	0	0	0	29	47	77
		M	B	1	0	0	1	12	59	73
	N588K	M	-	2	0	0	0	22	49	73
		M	B	7	2	0	0	22	44	75
32°C + N + S	N588D	M	-	6	0	0	0	31	56	93
		M	B	0	3	0	3	21	55	82
	N588K	M	-	1	0	1	0	34	44	80
		M	B	1	4	0	4	33	50	92

Supplementary Table 2 related to Fig. 2: Differentiation efficiencies.

	CMs (cTNT)										
	**409B2**	**SQT22**	**SQT96**	**SQT83**	**SQT87**	**LQT26**	**LQT46**	**LQT79**	**CTL1**	**CTL41**	**CTL60**
**RUN 1**	68.3	91.0	93.2	87.6	-	82.7	94.0	44.8	-	-	-
**RUN 2**	39.0	86.4	43.6	78.1	72.3	86.3	85.3	37.8	-	-	-
**RUN 3**	97.9	98.4	98.4	98.4	97.6	98.1	98.4	98.3	98.1	98.4	98.5
**RUN 4**	73.1	78.2	-	-	-	45.1	-	-	-	-	-
**AVE**	69.6	88.5	68.4	88.0	72.3	78.0	92.6	41.3	98.1	98.4	98.5
**STDEV**	24.2	8.5	35.1	10.2	-	22.9	6.7	4.9	-	-	-
	**MCs (CD90)**										
	**409B2**	**SQT22**	**SQT96**	**SQT83**	**SQT87**	**LQT26**	**LQT46**	**LQT79**	**CTL1**	**CTL41**	**CTL60**
**RUN 1**	-	-	-	-	-	-	-	-	-	-	-
**RUN 2**	97.0	85.4	90.8	84.3	82.0	84.2	71.5	50.4	-	-	-
**RUN 3**	98.6	98.6	-	99.0	-	98.8	93.4	-	-	-	-
**RUN 4**	99.4	98.6	-	-	-	99.6	-	-	-	-	-
**AVE**	98.3	94.2	90.8	91.6	82.0	94.2	82.4	50.4	-	-	-
**STDEV**	1.2	7.6	-	10.4	-	8.7	15.5	-	-	-	-

Supplementary Table 3 related to Fig. 3

	FPD (ms)	FPDcF (ms)	Beat Rate	Differentitation Run	CM percentage (%)	FCM day from differentiation day0	MEA plating day from differentiation day0	MEA record day from plating day	Average MEA record day from plating day
Parent	318	272	37	1st	68.3	17	17	4	4
	328	282	38						
	248	233	50						
	318	276	40						
	216	222	65	3rd	97.9	16	17	4	
	165	165	60						
	169	169	60						
SQT22	89	71	30	1st	91	17	17	4	5.5 ± 1.6
	75	57	26						
	91	72	31						
	94	79	35					5	
	82	67	33						
	72	59	33						
	99	97	57	2nd	86.4	18	18	5	
	94	89	52						
	99	93	49						
	117	114	55	3rd	98.6	16	17	8	
	106	102	53						
	93	88	51						
LQT26	375	308	33	1st	82.7	17	17	4	5.9 ± 2.0
	280	222	30						
	474	378	30						
	390	318	32						
	797	556	20	2nd	86.3	18	18	5	
	283	277	56	3rd	98.1	16	17	8	

	281	274	56						
	224	222	59						
	352	348	58						

Supplementary Table 4 related to Figure 4

	FPD (ms)	FPDcF (ms)	Beat Rate	Differentiation Run	Line	Mixed ratio (CM & MC)	3D CTS making day from day0	FCM day / MEA plating day from day0	MEA record day from plating day	Average MEA record day from plating day
Parent	249	241	55	3rd	Parent	1 to 1	20	24	13	10.7 ± 5.9
	213	214	61				20	24	17	
	323	290	43				20	24	20	
	238	187	29	4th			17	21	6	
	390	277	22				17	21	6	
	164	188	90				17	21	6	
	214	179	36				17	21	7	
SQT	112	108	54	3rd	SQT22		27	31	10	7.0 ± 1.7
	99	77	29	4th			17	21	6	
	104	82	30				17	21	6	
	100	85	36				17	21	6	
	102	83	32				17	21	7	
LQT	303	314	67	3rd	LQT46		27	31	3	8.4 ± 4.9
	322	334	67				27	31	10	
	267	329	112				27	31	16	
	392	356	45	4th	LQT26		17	21	6	
	401	267	18				17	21	7	

Supplementary Table 5 related to Supplementary Fig. 8: Field potential durations collected by Fridericia's formula and field potential durations after E-4031 treatment in 3D cardiac tissue sheets (±s.e.m.).

FPDcF	409B2 (n=3)	SQT22 (n=5)	LQT46 (n=3)
0 nM	249±22	86±12	326±6
0.3 nM	250±23	88±13	326±5
3 nM	262±23	91±13	336±2
10 nM	286±28	93±16	338±1
30 nM	323±25	100±24	334±4

FPD	409B2	SQT22	LQT46
0 nM	262±32	103±6	297±16
0.3 nM	263±33	105±7	297±14
3 nM	276±34	109±7	307±16
10 nM	302±39	111±8	307±18
30 nM	349±47	120±16	296±19

Supplementary Table 6 related to Supplementary Fig. 8: Beat rate after E-4031 treatment in 3D cardiac tissue sheets (±s.e.m.).

BR	409B2 (n=3)	SQT22 (n=5)	LQT46 (n=3)
0 nM	53±5	36±10	82±15
0.3 nM	53±5	36±11	82±15
3 nM	53±5	36±10	82±15
10 nM	52±5	35±11	83±16
30 nM	49±4	35±11	89±14

Supplementary Table 7: gRNA.

Target	Name	Sequence
KCNH2 c.1762A>G	KCNH2x1763	ATCGGCTGGCTGCACAACCT

Supplementary Table 8: ssODN templates.

Target	Name	Sequence
KCNH2 c.1762A>G	KCNH2-N588D-50/50-t	GGCCGCCCAGGCCGCTGCTGTTGTAGGGTTTGCCTATCTGGTCGCCCAGGTCGTGCAGCCAGCCGATGCGTGAGTCCATGTGTGGCTGCTCCATGTTGCC
KCNH2 c.1764C>A	KCNH2-N588K-50/50-t	GGCCGCCCAGGCCGCTGCTGTTGTAGGGTTTGCCTATCTGGTCGCCCAGTTTGTGCAGCCAGCCGATGCGTGAGTCCATGTGTGGCTGCTCCATGTTGCC
KCNH2 c.1765C>T	KCNH2-L589L-50/50-t	GGCCGCCCAGGCCGCTGCTGTTGTAGGGTTTGCCTATCTGGTCGCCCAAGTTGTGCAGCCAGCCGATGCGTGAGTCCATGTGTGGCTGCTCCATGTTGCC

Supplementary Table 9: Genotyping primers.

Target	Name	Sequence
KCNH2 c.1762A>G<br>KCNH2 c.1764C>A	dna2093-HERG-Exon8, 9-F	CTCTGTCCCAAAGCTAGCAC
	dna2094-HERG-Exon8, 9-R	GGGTCCTTACTACTGACTGTGA

Supplementary Table 10: Field potential durations collected by Fridericia's formula, Field potential durations, and Beat rate after DMSO treatment in 2D cardiomyocytes (±s.e.m) (n=5).

DMSO	FPDcF	FPD	Beat rate
0.10%	259±11	262±13	58±3
0.20%	258±11	261±11	58±2
0.30%	254±7	257±7	58±2
0.40%	252±9	253±10	59±3
0.50%	249±8	249±8	60±2

Supplementary Table 11: E-4031 concentration at the emergence of arrhythmia in 3D CTSs.

DMSO	Parent (n)	SQT (n)	LQT (n)
0.3 nM	1	1	0
3 nM	1	0	0
10 nM	1	0	0
30 nM	3	0	1
100 nM	1	0	3
1000 nM	5	7	1
